# Vancomycin-resistant *Enterococcus faecium* Clone in Swine, Europe

**DOI:** 10.3201/eid1112.050822

**Published:** 2005-12

**Authors:** Carla Novais, Teresa M. Coque, Patrick Boerlin, Inmaculada Herrero, Miguel A. Moreno, Lucas Dominguez, Luísa Peixe

**Affiliations:** * REQUIMTE at Universidade do Porto, Porto, Portugal; † Hospital Universitario Ramón y Cajal, Madrid, Spain; ‡ University of Guelph, Ontario, Canada; § Universidad Complutense de Madrid, Madrid, Spain

**Keywords:** vancomycin resistance, Europe, clone, E. faecium, swine, Portugal, Spain, Switzerland, Denmark, letter

**To the Editor:** The use of antimicrobial agents for growth promotion (AGP) in food-producing animals has been extensively debated because of the risk of establishing a reservoir of antimicrobial resistance genes or antimicrobial-resistant organisms of potential relevance for human health. This concern has motivated the progressive ban of the use of different AGP in the European Union, which began in 1997 with avoparcin and will end in 2006 (*1*). Worldwide trade of living animals for food production or breeding and of meat products enables multidrug-resistant pathogens to spread across national borders.

Intercontinental dissemination of antimicrobial-resistant bacteria associated with food animals has been described for particular clones such as *Salmonella enterica* Typhimurium DT104 or *Escherichia coli* O157:H7 and for transferable genetic elements such as the genomic island SG1 or the streptococcal plasmid pRE25 (*2*). Vancomycin-resistant enterococci (VRE) in European farms were initially associated with the intensive use of avoparcin; however, the persistence of VRE in food animal environments after years of avoparcin withdrawal indicates that coselection by further antimicrobial or other agents, increased fitness of strains, and mobile genetic elements cannot be ruled out (*1**–**3*).

A specific clone was recently detected among vancomycin-resistant *E. faecium* (VREF) isolated from different swine farms in Denmark and Switzerland and from a healthy Danish woman without antimicrobial drug exposure who ate pork, chicken, and beef (*4**,**5*). Since Portugal and Spain maintain commercial trade of food-producing swine (living or meat products) between them and with other European countries, including Denmark (http://www.dgv.min-agricultura.pt/dgv.nsf), we investigated a possible relationship among VREF swine fecal isolates from Portugal and Spain and compared these isolates with the Swiss/Danish clone. We studied 3 VREF from a Figueira da Foz slaughterhouse in central Portugal (1997–1998) and 3 VREF isolates from 3 Spanish slaughterhouses in Valencia, Lugo, and Murcia in eastern, northern, and southern Spain, respectively (1998–2000). These isolates were recovered in the course of previous surveillance studies (C. Novais/I. Herrero, unpub data). Antimicrobial susceptibility was tested for 13 antimicrobial agents by using the agar dilution method (*6*). Clonal relationships were analyzed by pulsed-field gel electrophoresis (PFGE) and characterization of *pur*-K alleles by amplification and further sequencing (6,7; http://efaecium.mlst.net). Species identification, genes coding for antimicrobial resistance genes or for putative virulence traits, and the backbone structure of Tn*1546* were analyzed by polymerase chain reaction followed by sequencing when necessary (*6**,**8*). Broth and filter mating were performed by using *E. faecium* GE1 as recipient strain (*6*).

Following criteria published elsewhere (*6*), the VREF isolates studied were considered a single clone (0–4 bands difference by PFGE). Some vancomycin-susceptible *E. faecium* swine isolates (VSEF) from Spain and Switzerland showed an *Sma*I-PFGE pattern closely related to that of VREF isolates (data not shown [4]; ).

Representative VREF of each country harbored the allele 9 of the housekeeping gene *pur*K, previously found among *E. faecium* isolates from swine and healthy persons (*7*). All VREF isolates were resistant to glycopeptides (*vanA*), erythromycin [*erm*(B)], and tetracycline. Two Spanish isolates were also highly resistant to streptomycin and kanamycin [*aph*(*3´*)-*IIIa*] (Table). All VREF isolates tested carried a Tn*1546* type D, previously found in isolates from food-producing animals (*8*). This element showed alterations in *orf1* and a G-T point mutation in the position 8234 at *van*X. Transfer of vancomycin resistance was detected for the Swiss (*4*), Spanish, and Portuguese isolates and was associated with erythromycin resistance in all cases. Tetracycline resistance was also transferable in the Spanish strains. No virulence traits were detected.

**Table:** Features of vancomycin-resistant Enterococcus faecium swine isolates from European countries*

Isolate	PFGE	purK	Antimicrobial-drug susceptibility (mg/L)*†													Resistance genes†	Mating‡
			VC	TC	AMP	TET	ER	CP	CL	GM	KM	SM	LIN	DA	NIT		

Portugal§																	
7S4	A	9	>256	256	<2	64	32	<0,5	8	<256	1,000	<256	2	1	64	*vanA* *erm*(B)	10^–4^
8S1	A3	ND	>256	256	<2	32	>32	<0,5	16	<256	<256	<256	2	1	64	*vanA* *erm*(B)	10^–8^
35S2	A4	ND	>256	256	<2	64	>32	<0,5	8	<256	<256	<256	2	0.5	32	*vanA* *erm*(B)	ND
Spain¶																	
S1	A1	9	>256	128	<2	>64	>32	<0,25	8	<256	>2,000	>2,000	2	1	64	*vanA* *erm*(B) *aphIII*	10^–5^
S2	A2	ND	>256	128	<2	64	>32	<0,25	8	<256	<256	<256	2	2	64	*vanA* erm(B)	10^–4^
S8	A1'	ND	>256	128	<2	>64	>32	<0,25	8	<256	>2,000	2,000	2	0.5	64	*vanA* *erm*(B) *aphIII*	10^–5^
Switzerland#																	
4D	A	9	>256	64	<2	64	>32	<0,25	<4	<256	<256	<256	2	4	64	*vanA* *erm*(B)	10^–5^

*PFGE, pulsed-field gel electrophoresis; VC, vancomycin; TC, teicoplanin; AMP, ampicillin; TET, tetracycline; ER, erythromycin; CP, ciprofloxacin; CL, chloramphenicol; GM, high level of resistance to gentamicin; KM, high-level resistance to kanamycin; SM, high-level resistance to streptomycin; LIN, linezolid; DA, daptomycin; NIT, nitrofurantoin; ND, not done. All isolates were TN*1546* type D.

†Antimicrobial resistance or resistance genes detected in transconjugants appear underlined.

‡Conjugation frequency is expressed as transconjugants per donors.

§First 2 isolates were collected in 1997; third in 1998.

¶S1 was isolated in 1998; S2, 1999; and S8, 2000.

#Isolate was collected in 1999.

We describe the simultaneous occurrence of a VREF strain among swine in 4 distant European countries for at least a 4-year period. Tn*1546* type D has been largely described in European swine isolates, which indicates stability of this particular type among the high diversity of Tn*1546* described to date (*8*). The finding of a group of genetically closely related strains, which include both VSEF and VREF isolates and which harbor a particular *pur*K allele previously associated with *E. faecium* swine strains, might mirror wide dissemination of a host-specific clone more prone than others to acquire and spread different antimicrobial resistance, as reported for human clinical *E. faecium* isolates (*9*). Since enterococci from swine are able to colonize in the human gut (*5**,**7*) and isolates harboring *pur*K-9 can be recovered from hospitalized patients with severe infections (*10*), specific swine enterococcal strains might represent a risk for antimicrobial resistance spread in the clinical setting. Further analyses need to be performed to understand the role of international animal movements, animal feed, and colonized farmers in the spread of this particular strain and to assess whether this clone shows an increased fitness in the porcine intestine when compared to other *E. faecium* strains.
